# Gluconate 5-dehydrogenase (Ga5DH) participates in *Streptococcus suis* cell division

**DOI:** 10.1007/s13238-014-0074-8

**Published:** 2014-06-11

**Authors:** Zhongyu Shi, Chunling Xuan, Huiming Han, Xia Cheng, Jundong Wang, Youjun Feng, Swaminath Srinivas, Guangwen Lu, George F. Gao

**Affiliations:** 1 CAS Key Laboratory of Pathogenic Microbiology and Immunology, Institute of Microbiology, Chinese Academy of Sciences, Beijing, 100101 China; 2 University of Chinese Academy of Sciences, Beijing, 100049 China; 3 College of Animal Science and Technology, Shanxi Agricultural University, Taigu, 030801 China; 4 Department of Microbiology, University of Illinois, Urbana, IL 61801 USA; 5 Department of Biochemistry, University of Illinois, Urbana, IL 61801 USA; 6 Laboratory of Protein Engineering and Vaccines, Tianjin Institute of Industrial Biotechnology, Chinese Academy of Sciences, Tianjin, 300308 China; 7 Research Network of Immunity and Health (RNIH), Beijing Institutes of Life Science, Chinese Academy of Sciences, Beijing, 100101 China

**Keywords:** *Streptococcus suis*, Ga5DH, cell shape, cell division, FtsZ localization

## Abstract

Bacterial cell division is strictly regulated in the formation of equal daughter cells. This process is governed by a series of spatial and temporal regulators, and several new factors of interest to the field have recently been identified. Here, we report the requirement of gluconate 5-dehydrogenase (Ga5DH) in cell division of the zoonotic pathogen *Streptococcus suis*. Ga5DH catalyzes the reversible reduction of 5-ketogluconate to D-gluconate and was localized to the site of cell division. The deletion of Ga5DH in *S. suis* resulted in a plump morphology with aberrant septa joining the progeny. A significant increase was also observed in cell length. These defects were determined to be the consequence of Ga5DH deprivation in *S. suis* causing FtsZ delocalization. In addition, the interaction of FtsZ with Ga5DH *in vitro* was confirmed by protein interaction assays. These results indicate that Ga5DH may function to prevent the formation of ectopic Z rings during *S. suis* cell division.

## INTRODUCTION

*Streptococcus suis* (*S. suis*) is an emerging zoonotic pathogen that causes life-threatening diseases, including septicemia, meningitis, and endocarditis (Kay et al., 1995; Mazokopakis et al., 2005), with more than 750 reported human cases of infection worldwide (Feng et al., 2010). Amongst the 35 known serotypes of *S. suis,* serotype 2 (SS2) is considered to be the most pathogenic and prevalent form in both pigs and humans (Nghia et al., 2008). In addition to sporadic cases of human SS2 infections (Feng et al., 2009; Feng et al., 2010), two large scale outbreaks of human SS2 endemics, with an unprecedented high rate of morbidity and mortality in China, were reported in 1998 and 2005 (Tang et al., 2006). Potent inhibitors and therapeutic agents to effectively control *S. suis* infection are required to address the re-emergence of SS2 as a zoonotic pathogen in humans and the rapid increase of antibiotic-resistant strains among clinical isolates.

D-gluconate, an important carbon source for many microorganisms, is required for *Escherichia coli* (*E. coli*) to colonize the streptomycin-treated mouse large intestine (Sweeney et al., 1996), suggesting that gluconate might play an important role in both bacterial survival and virulence. Membrane-bound gluconate 5-dehydrogenase (Ga5DH) catalyzes the inter-conversion of D-gluconate and 5-keto-D-gluconate, while simultaneously generating NADPH, which acts as a hydrogen donor for many biosynthetic processes. Cell cycle proteins have traditionally been an attractive target for antibacterial agents, but with our increasing understanding in this area, Ga5DH also seems like an effective target for the development of novel potent antibacterial agents. In our previous work, *S. suis* Ga5DH was characterized both structurally and enzymatically (Zhang et al., 2009).

Cell division is initiated by the formation of a cytokinetic ring at the prospective division site (Bi & Lutkenhaus, 1991), which leads to the production of two identical daughter cells. Ellipsoid-shaped bacteria such as *S. suis* divide at the midpoint of the cell along successive parallel planes perpendicular to the long axis. The first known event in bacterial cytokinesis is the polymerization of the tubulin homologue GTPase FtsZ into a ring structure at the prospective site of division (Bi and Lutkenhaus, 1991). The polymerization of FtsZ into the Z ring at the future division site is critical for cell division because it guides septum synthesis, location, and shape (Addinall & Lutkenhaus, 1996). Diverse cell division components interact with FtsZ to regulate FtsZ assembly. In *E. coli*, two division proteins, ZipA and FtsA, directly interact with FtsZ and cooperate in anchoring FtsZ to the membrane (Pichoff & Lutkenhaus, 2002).

Bacteria display a wide variety of cell shapes. Although some divisome proteins such as FtsZ are conserved among nearly all bacteria, other components of the cell division machinery diverge significantly to reflect the diversity of bacterial shapes. For example, the Min proteins are required for preventing Z ring assembly at cell poles in *Bacillus subtilis* and *E. coli* but are missing from some cocci, such as *Staphylococcus*, *Enterococcus*, and *Streptococcus* (Zapun et al., 2008).

The mechanism of FtsZ localization and the regulators that affect Z ring assembly have been extensively studied in the rod-shaped laboratory workhorses *E. coli* and *B. subtilis*. In this study, we report for the first time that Ga5DH plays a role in the cell growth and cell division of *S. suis*. In the absence of Ga5DH, cells exhibited a reduced growth rate and plump sausage-like shape with non-constricted septa joining the progeny. We found that the cells lacking Ga5DH display aberrant formation of FtsZ rings. Furthermore, protein interaction studies revealed that Ga5DH is capable of binding to FtsZ *in vitro*. These results suggest that Ga5DH is involved in maintaining correct cell shape and in cell division.

## RESULTS

### Construction of Δ*ga5dh* in *S. suis* 05ZYH33

**Figure 1:**
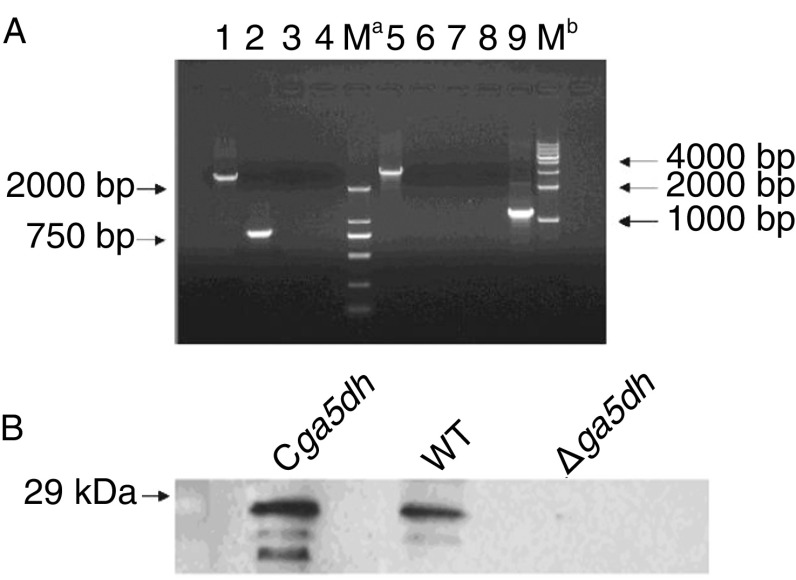
**Construction and confirmation of the Δ** ***ga5dh*** **mutant** ***S***. ***suis*** **and its complemented strain by multiplex PCR and Western blot analyses**. (A) Genomic DNA extracts from the WT or the deletion mutant were used as templates: WT strain (lanes 1–4) and Δ*ga5dh* mutant (lanes 5–9). Two different DNA ladder markers, which are marked as M^a^ and M^b^ respectively, were used and labeled. The primer pairs and the theoretical size (bp) of the indicated PCR product are as follows: lanes 1: LU/RD, 2892 bp; 2: *ga5dh*-F/*ga5dh*-R, 835 bp; 3: *ga5dh*-F/spc-R, (-); 4: spc-F/*ga5dh*-R, (-); 5: LU/RD, 3209 bp; 6: *ga5dh*-F/*ga5dh*-R, (-); 7: *ga5dh*-F/spc-R, (-); 8: spc-F/*ga5dh*-R, (-); and 9: spc-F/spc-R, 1130 bp. (B) Western blot assay characterizing the protein expression of Ga5DH. The indicated *S. suis* cells were probed with Ga5DH polyclonal antibodies, and the expression profiles are shown

SS2 05ZYH33 was isolated from patients with streptococcal toxic shock syndrome (STSS) in Sichuan Province, China (Tang et al., 2006). To further investigate the function of Ga5DH in the bacterial physiology of SS2, we constructed a homologous suicide plasmid, pUC::*ga5dh*, with a *spcR* cassette. The suicide plasmid was then transformed into *S. suis*, and positive transformants were screened on THY agar plates with the selective antibiotic spectinomycin. Successful construction of the Δ*ga5dh* strain was confirmed by multiplex-PCR analysis. In the Δ*ga5dh* mutant, the entire *ga5dh* gene was replaced with a spectinomycin cassette (Fig. 1A). Western blot assays confirmed the absence of Ga5DH in the deletion mutant and the return of Ga5DH expression in the complemented strain (Fig. 1B).

### Growth phenotype of the Δ*ga5dh* mutant

**Figure 2:**
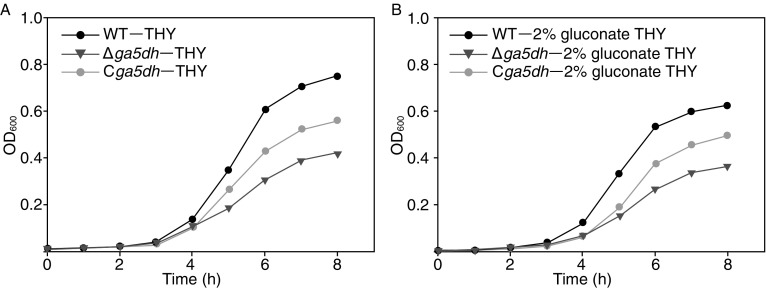
**Ga5DH is required for** ***S***. ***suis*** **growth**. Comparative analyses of growth curves from three *S. suis* strains (WT, the Δ*ga5dh* mutant, and the complemented strain C*ga5dh*). The strains were grown in THY liquid medium (A) or in THY liquid medium supplemented with 2% (*w*/*v*) gluconate (B). Each point represents the mean ± SD OD_600_ value from triplicate experiments

We first characterized the growth kinetics of the mutant strain. The wild type (WT) and Δ*ga5dh* strains were grown overnight and then inoculated into fresh nutrient-rich THY medium at 37°C. A reduced growth rate was observed for the Ga5DH-deficient cells compared to the WT strain, indicating that Ga5DH is critical for *S. suis* growth (Fig. 2A). Indeed, complementation of *ga5dh* (C*ga5dh*) partially restored the growth defect. It is noteworthy that in THY medium, where gluconate is not utilized as the main carbon source, the Δ*ga5dh* growth defect phenotype was still dramatic.

We further examined the cell growth of the WT, deletion mutant, and complemented strains in THY medium with extra carbon source by supplementation with 2% (*w*/*v*) gluconate sodium (Fig. 2B). Similar growth profiles to those obtained in THY medium were recorded. Based on these observations, we believe it is unlikely that the impaired cell growth in Δ*ga5dh* was due to an energy deficiency or exhaustion. Therefore, we hypothesized that Ga5DH might function to affect *S. suis* cell division, in addition to its traditional role as a gluconate metabolic enzyme.

### Deletion of the *ga5dh* gene affects cell morphology and division

**Figure 3:**
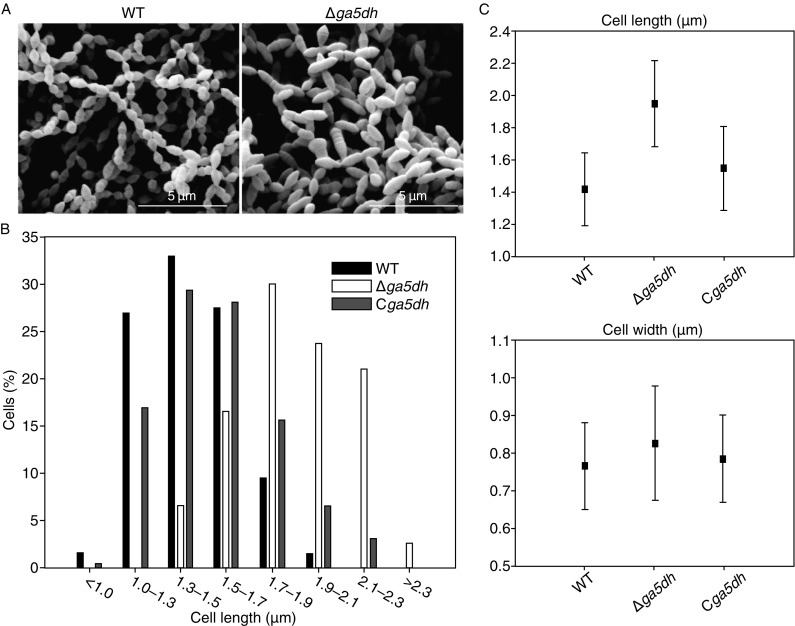
**Micrographs of WT, Δ** ***ga5dh*** **mutant cells, and C** ***ga5dh*** **cells**. (A) Scanning electron micrographs of the WT and the Δ*ga5dh* mutant strains. (B) Histogram statistics of the WT (*n* = 200, black), Δ*ga5dh* (*n* = 200, white), and C*ga5dh* (*n* = 200, gray) cell lengths after growth in THY medium. To determine the cell length, we measured the longitudinal length of single membrane-stained cells. Δ*ga5dh* cells were significantly longer than WT cells. (C) Cell size parameters of the WT, Δ*ga5dh*, and C*ga5dh* cells. The cell length of Δ*ga5dh* cells was significantly different from that of the WT strain (*P* < 0.01; two-tailed *t*-test)

Scanning electron microscopy (SEM) was employed to study the morphology and division pattern of the WT and mutant *S. suis* strains. WT cells grew as diplococci or short chains showing a characteristic ellipsoid shape and normal division pattern. The Δ*ga5dh* strain, however, developed into plump, sausage-shaped cells that were less ovoid and significantly longer than the WT cells. In addition, multiple indentations appeared on the cell surface of the Δ*ga5dh* strain (Fig. 3A). A quantitative analysis of the cell length was further performed to analyze the morphological phenotypes of the *ga5dh* deletion mutant (Fig. 3B and 3C). Single membrane-stained cells were randomly selected, and their longitudinal length was measured. The statistics of cell length for the three *S. suis* strains (WT, Δ*ga5dh*, and complemented) all revealed a typical Gaussian distribution pattern. Nevertheless, the WT and the complemented bacteria concentrate mainly in a length interval of 1.0–1.7 μm, whereas the majority of the Δ*ga5dh* cells were distributed in a region with a cell length of 1.5–2.3 μm (Fig. 3B). On average, the calculated cell length along the long axis in the deletion strain was 1.95 μm (*n* = 200), compared to 1.42 μm in the WT cells (*n* = 200) and 1.55 μm in the Ga5DH-complemented cells. Cell width in Δ*ga5dh* cells was also slightly greater than that of the WT cells (Fig. 3C).

**Figure 4:**
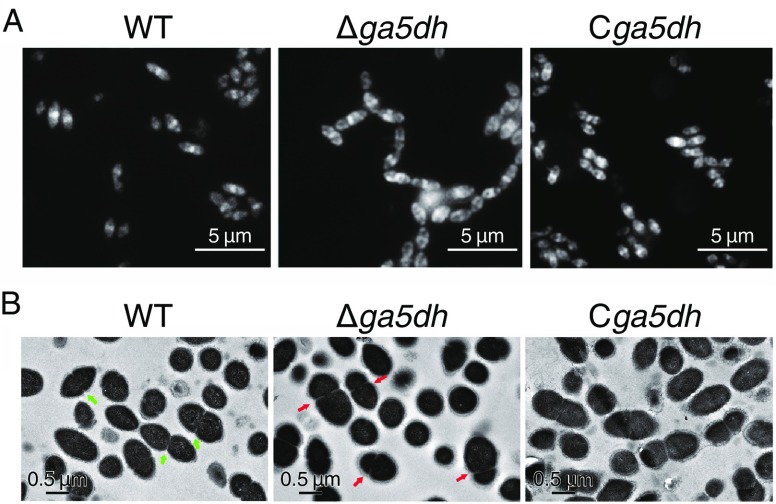
**Micrographs of the WT, Δ** ***ga5dh*** **, and C** ***ga5dh*** **strains**. (A) FM4-64 staining; scale bar, 5 μm. (B) Transmission electron micrographs; scale bar, 0.5 μm. Green arrows highlight the correct septum placement in WT cells. Red arrows mark the aberrant septum placement in Δ*ga5dh* cells. Cell morphology of the WT strain revealed the characteristic ellipsoid shape and normal division pattern of *S. suis*, with correct septum placement and symmetric daughter cells. The mutant cells displayed an increased length and multiple aberrant septa with incorrect placement

Additionally, we used transmission electron microscopy (TEM) and FM4-64 (a styryl membrane-specific dye) staining to gain an exact view of the cell shape and cell septa of both the WT and the mutant cells in details. In contrast to the WT cells with their typical ovoid shape, Δ*ga5dh* cells were enlarged, and their cell poles were round (Fig. 4A and 4B). Further, by staining with FM4-64, severe defects in morphology were observed in the Δ*ga5dh* cells. For example, the mutant cells were significantly longer and presented multiple aberrant septa. Cell constriction was also impaired, indicating that the septa might have no or abnormal functions. In addition, the Δ*ga5dh* cells exhibited severe division defects, including abnormal septum position (Fig. 4A) and asymmetrical divisions (Fig. 4B). For the WT strain, however, the dividing cells displayed the normal tight coordination of septum formation and constriction, showing characteristics of a symmetrical division. As expected, complementation of the *ga5dh* gene largely restored a normal cell-dividing phenotype in *S. suis*. These data indicate that Ga5DH is involved in *S. suis* cell division and suggest that Ga5DH might play a role in septum constriction.

### Distribution of Ga5DH at mid-cell sites

**Figure 5:**
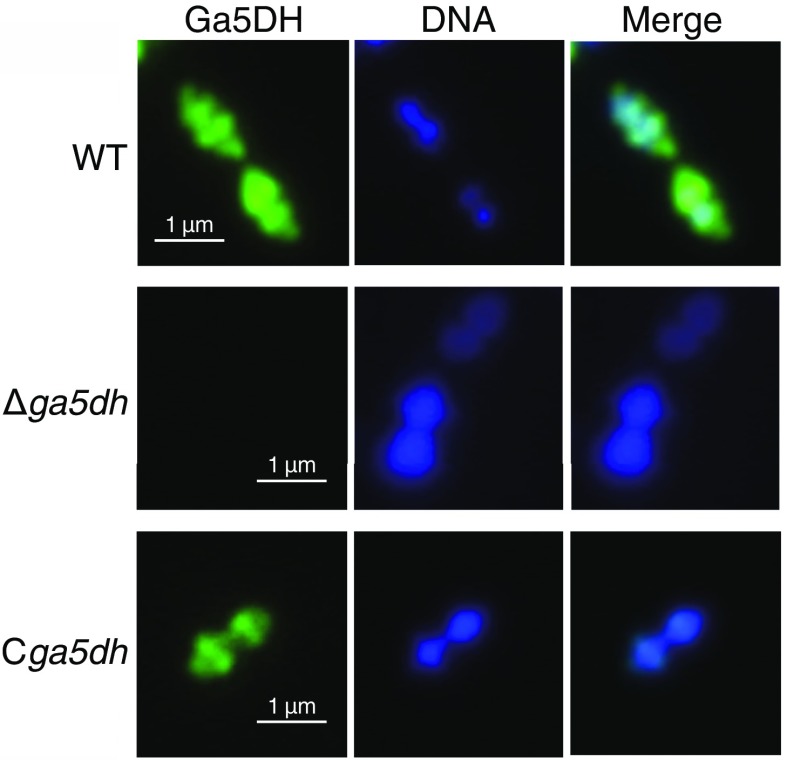
**Subcellular localization of Ga5DH**. DNA was visualized with DAPI (blue). Ga5DH was visualized using an anti-Ga5DH polyclonal antibody and an anti-mouse IgG secondary antibody coupled to Alexa Fluor 488 (green). The merged pictures show the overlay of Ga5DH and DAPI staining. Scale bar, 1 μm

We further tested the subcellular localization of Ga5DH in exponentially growing cells by fluorescence microscopy using polyclonal antibodies directed against Ga5DH. As expected, no signal was detected in the Δ*ga5dh* cells (Fig. 5). However, the Ga5DH protein in both the WT and the complemented cells was mainly distributed as bands at the cell division septum (Fig. 5).

### Ga5DH interacts with FtsZ *in vitro*

**Figure 6:**
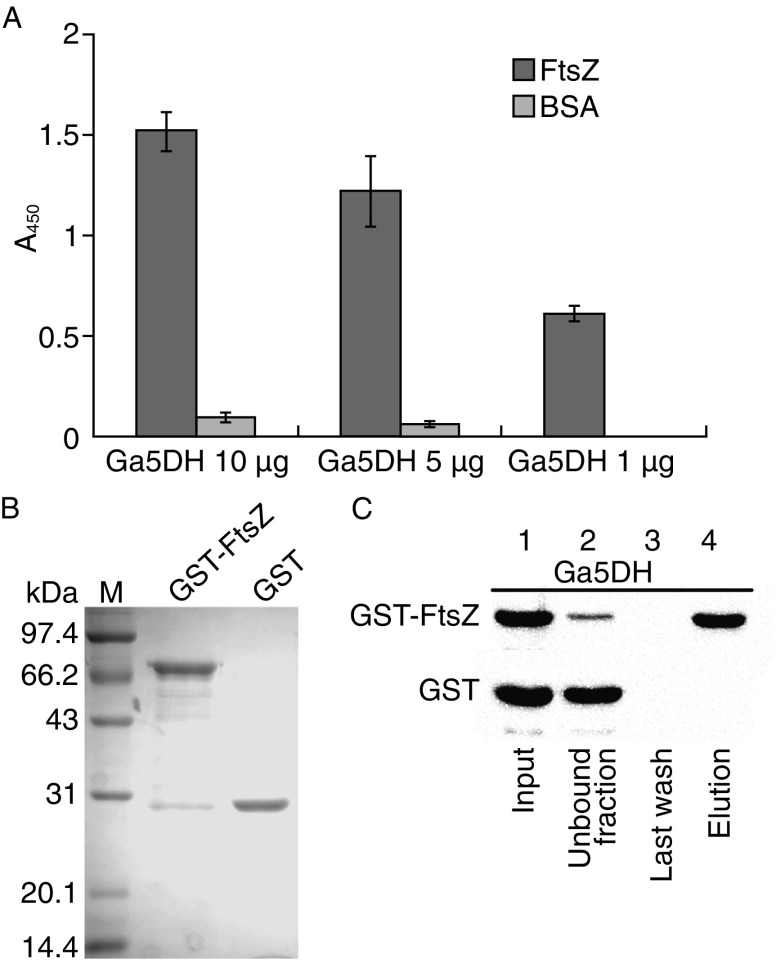
**Ga5DH interacts with FtsZ** ***in vitro***. (A) Solid-phase binding assay for FtsZ and Ga5DH. Wells of a microtiter plate were coated with FtsZ or BSA and incubated with various concentrations of Ga5DH protein as indicated. The bound Ga5DH protein was immunodetected with Ga5DH polyclonal antibodies by ELISA. (B) Inputs of GST-FtsZ and GST in the GST pull-down assay detected by SDS-PAGE. (C) GST pull-down assay characterizing the interaction between FtsZ and Ga5DH. Lane 1: the input His-tagged Ga5DH in the pull-down assay. Lane 2: the unbound fraction of Ga5DH. Lane 3: the last wash of the unbound fraction of Ga5DH. Lane 4: the eluted protein fraction using 20 mmol/L glutathione. All samples were detected by Western blotting using an anti-His monoclonal antibody

The subcellular localization of Ga5DH at the cell division septum implied a potential interaction of Ga5DH with FtsZ, an important cell division initiation factor that also localizes at this site. A variety of assays were performed to evaluate the physical interactions between the Ga5DH and FtsZ proteins of *S. suis in vitro*. First, FtsZ- or BSA-coated microtiter enzyme-linked immunosorbent assay (ELISA) plates were incubated with varying concentrations of Ga5DH. Following washing, the bound Ga5DH protein was immunolabeled and quantified by ELISAs. As shown in Fig. 6A, Ga5DH bound to the FtsZ-coated wells but not to the wells coated with BSA.

Next, GST pull-down assays were performed to further confirm the interaction between Ga5DH and FtsZ. FtsZ was N-terminally fused to GST, expressed in *E. coli*, and purified to homogeneity (Fig. 6B). To determine whether Ga5DH and FtsZ could form a stable complex, equimolar amounts of GST-FtsZ or GST were mixed with *E. coli* extracts containing Ga5DH and loaded onto a column packed with glutathione resin. Following washing, the bound proteins were eluted with glutathione. Bound Ga5DH, which was identified by Western blotting, was detected only for GST-FtsZ but not for the control protein of purified GST (Fig. 6C). Therefore, we provide solid evidence that Ga5DH directly interacts with FtsZ.

### Deletion of Ga5DH results in Z ring delocalization

**Figure 7:**
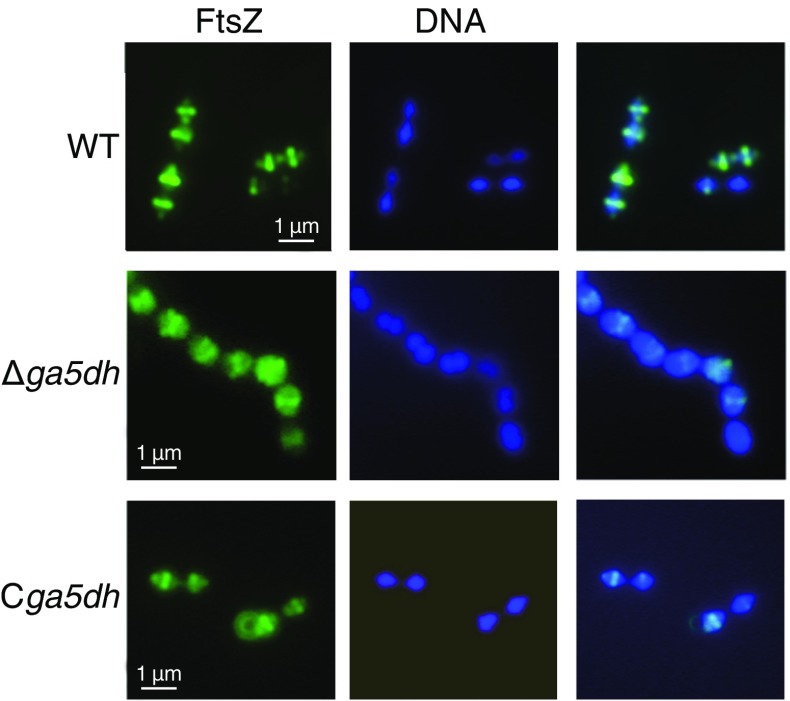
**Delocalization of FtsZ rings in** ***S***. ***suis*** **cells lacking Ga5DH**. The morphology of FtsZ was detected by immunolabeling with anti-FtsZ polyclonal antibodies and an anti-mouse IgG secondary antibody coupled to Alexa Fluor 488 (green). DNA was visualized with DAPI (blue). The merged pictures show the overlay of FtsZ and DAPI staining. Scale bar = 1 μm

The *in vitro* observation of a direct Ga5DH/FtsZ interaction urged us to further analyze the effect of Ga5DH deletion on FtsZ localization *in vivo*. Immunofluorescence assays of septal FtsZ ring morphology in the WT and Δ*ga5dh* cells were performed using a polyclonal antibody against FtsZ. As expected, the majority of WT cells displayed FtsZ protein that was regularly distributed as a line at mid-cell (Fig. 7). The FtsZ localization in the Δ*ga5dh* cells, however, was dramatically altered compared to that in the WT cells. For the Ga5DH deletion mutant, FtsZ was present in aberrant singlets or doublets, distributed along the length of the cell. Moreover, in the absence of Ga5DH, some FtsZ failed to localize to the potential division sites. As expected, complementation of the *ga5dh* gene faithfully restored a normal FtsZ localization pattern (Fig. 7). This suggests that in addition to its enzymatic activity, Ga5DH may play a physical role in *S. suis* cell division for Z ring localization.

## DISCUSSION

Tight regulation of the cell wall synthesis and degradation machinery is required to maintain the proper shape and size of bacterial cells. Septal cell wall formation during division must be coordinated with other processes of cell division, such as membrane invagination, DNA replication, and chromosome segregation. To avoid a catastrophic breakage of the chromosome during division, cell separation must occur exactly at the middle of the cell to ensure the generation of two equal daughter cells. It is known that the initiation of cell division requires the proper location of the Z ring at mid-cell. Here, our data indicate that Ga5DH, a gluconate metabolic enzyme, has an important role in *S. suis* cell division.

The deletion of *ga5dh* led to aberrant cell shape and division. Compared to the WT cells, the deletion mutant exhibited increased length, with non-constricted septa, reminiscent of the phenotypes exhibited by *pneumococcus* *stkP-KD-TMH* mutant cells (Fleurie et al., 2012). Fleurie et al. confirmed that the misplacement of StkP abolishes its function and affects cell division. The same cell morphology was also observed in *pneumococcus* cells with PBP2b^28^ (a mutant PBP2b sequence obtained from the *Streptococcus pneumoniae* Cba-28 clinical strain) proteins, which display a rod-like shape (Albarracin Orio et al., 2011). TEM revealed that the rod-shaped cells exhibit multiple septa, and it was proposed that the direct interaction of PBP2b^28^ with FtsZ leads to the mislocalization of FtsZ and to an aberrant cellular morphology. Here, we describe plump, sausage-shaped cells and an abnormal cell division pattern caused by the deletion of Ga5DH. Moreover, we confirmed that Ga5DH localized mainly at the septum and directly interacted with the essential cell division initiation factor FtsZ. In addition, the deletion of Ga5DH altered the normal mid-cell localization of FtsZ. These observations indicate a role of Ga5DH in the FtsZ localization at the septal site, though it remains to be investigated whether Ga5DH has a direct or an indirect impact on FtsZ assembly and subsequent cell division.

The primary role of Ga5DH is metabolic, catalyzing the inter-conversion of D-gluconate and 5-keto-D-gluconate. In previous studies, other metabolic proteins such as ManA and UgtP have been reported to affect cellular structures and cell division in *B. subtilis* (Elbaz & Ben-Yehuda, 2010; Weart et al., 2007). In *B. subtilis,* nutrient availability has a dramatic effect on UDP-glucose accumulation. Under conditions in which UDP-glucose levels are high, UDP-glucose directly interacts with the diacylglycerol glucosyltransferase UgtP to inhibit FtsZ assembly and delay maturation of the Z ring. It is possible that the metabolic enzyme Ga5DH operates as a sensor that synchronizes cell division with metabolite availability through its role in coordinating FtsZ localization.

## MATERIALS AND METHODS

### Bacterial strains, plasmids, and culture conditions

Strains and plasmids used in this study are listed in Table 1. *Streptococcus suis* strains were grown in Todd-Hewitt broth (THB) (Difco Laboratories, Detroit, MI, USA) supplemented with 2% yeast extract (THY) or plated on THY agar at 37°C (Feng et al., 2007; Feng et al., 2008). THY medium and THY medium supplemented with 2% gluconate were used for growth curve measurements. Experiments were performed in triplicate.

**Table 1:** Bacterial strains and plasmids used in this study

Strains/ plasmids	Characteristics	Origins
Bacterial strains		
05ZYH33	A virulent Chinese isolate of *S. suis* serotype 2	Feng et al. (2008); Tang et al. (2006)
05ZYH33Δ*ga5dh*	05ZYH33 derivative with the *ga5dh* gene replaced by a spcR gene cassette	This work
C*ga5dh*	Complemented strain of 05ZYH33 Δ*ga5dh*	This work
DH5α	An *E. coli* cloning host	Lab stock
BL21	An *E. coli* expression host	Lab stock
Plasmids		
pUC-18	Cloning vector	TaKaRa
pET28b	His-tag fusion expression vector	Novagen
pET30a	His-tag fusion expression vector	Novagen
pGEX-6P-1	GST-tag fusion expression vector	Novagen
pVA-838	*E. coli* - *S. suis* shuttle vector	Lab stock

*E. coli* strains DH5α and BL21 were incubated in Luria-Bertani (LB) medium or plated on LB agar. When required, antibiotics were routinely added at the following concentrations: spectinomycin, 100 mg/mL for both *S. suis* and *E. coli*; erythromycin, 1 mg/mL for *S. suis* and 250 mg/mL for *E. coli*. 50 mg/mL of ampicillin was always present to screen the transformants of *E. coli*.

### Cloning, mutagenesis, and genetic complementation

To delete *ga5dh*, the two flanking sequences of *ga5dh* were amplified from the chromosomal DNA of *S. suis* 05ZYH33 (Feng et al., 2008; Li et al., 2008; Li et al., 2011). All primers used in this study are listed in Table 2. For cloning *ga5dh* homologous regions, two pairs of specific primers (LA-P1/LA-P2 and RA-P1/RA-P2) carrying *Eco*RI/*Bam*HI and *Pst*I/*Hin*dIII restriction enzyme sites were used, respectively. After digestion with the corresponding restriction enzymes, the DNA fragments were cloned into a pUC18 vector to generate the recombinant plasmid pUC::*ga5dh*LR. Then, the *spcR* gene cassette was inserted into *Bam*HI/*Pst*I digested plasmid pUC::*ga5dh*LR to generate the *ga5dh* knockout plasmid pUC::*ga5dh*. To obtain the isogenic mutant Δ*ga5dh*, *S. suis* 05ZYH33 was transformed by electroporation of the resulting plasmid pUC::*ga5dh* following previously described procedures (Feng et al., 2008; Li et al., 2008).

**Table 2:** Primers used in this study

Primers	Sequence (5′–3′)	Function
LU	TTCCGGCTGTTTACCAATGT	PCR detection
RD	CTAAAGGGAAAAATTGCTTTG	
LA-P1 (*Eco*RI)	CCGGAATTCATAGCTTCCAAACTAGACTGG	Mutant strain construction
LA-P2 (*Bam*HI)	CGCGGATCCATAAGAAGGGAAAAAACGATG	
RA-P1 (*Pst*I)	AACTGCAGATTCGTAACTCCCTTGTATTT	Mutant strain construction
RA-P2 (*Hin*dIII)	CCCAAGCTTTTGTTTTCATGTACACTTTTG	
C*ga5dh*-F (*Bam*HI)	CGCGGATCCCTGATATGACTATATAAAATG	*ga5dh* cloning and complementation
C*ga5dh*-R (*Sal*I)	GCGTCGACTTACGCTTCCGGCTGTTTA	
spc-F (*Bam*HI)	GCAGGATCCGTTCGTGAATACATGTTATA	*spc* cloning and PCR detection
spc-R(*Pst*I)	GGCTGCAGGTTTTCTAAAATCTGAT	
*ftsZ*-F (*Nde*I)	GGAATTCCATATGGCATTTTCATTTGAAGCA	*ftsZ* cloning into pET30a and protein expression
*ftsZ*-R (*Xho*I)	CCGCTCGAGGCGATTACGGAAGAATGG	
*ftsZ*-GST-F(*Bam*HI)	CGCGGATCCATGGCATTTTCATTTGAAGCA	*ftsZ* cloning into pGEX-6P-1 and protein expression
*ftsZ*-GST-R(*Eco*RI)	CCGGAATTCTTAGCGATTACGGAAGAATGG	
*ga5dh*-F (*Nde*I)	GGAATTCCATATGAATCAGCAATTTTC	*ga5dh* cloning in pET28b and protein expression (Zhang et al., 2009)
*ga5dh*-R (*Xho*I)	CCGCTCGAGTTACGCTTCCGGCTGTTT	

The underlined sequences represent the restriction sites

To construct the complement strain, a DNA fragment encoding *ga5dh* and its promoter region were generated by PCR using specific primers (C*ga5dh*-F/C*ga5dh*-R) and inserted into the digested *E. coli*-*S. suis* shuttle vector pVA838 (Macrina et al., 1982). And then the resulting plasmid pVA*::ga5dh* was transformed by electroporation into the Δ*ga5dh* mutant. Transformants were screened on THY plates with selection for spectinomycin and erythromycin resistance.

### Overexpression and purification of FtsZ and Ga5DH

The *ftsZ* gene was amplified by PCR using the chromosomal DNA of *S. suis* 05ZYH33 as template and specific primers described in Table 2. The *ftsZ* PCR product was digested by enzymes *Nde*I and *Xho*I and then inserted into the digested pET30a vector to generate the recombinant plasmid pET30-ftsZ. For expression of FtsZ with a fused GST tag, *ftsZ* gene was cloned into the pGEX-6P-1 vector via the *Bam*HI and *Eco*RI sites. For expression of His-tagged Ga5DH, the *ga5dh* PCR product was inserted into the pET28b vector via the *Nde*I and *Xho*I restriction sites (Zhang et al., 2009). The resulting recombinant plasmid was termed pET28b-ga5dh. In each case, the recombinant proteins were over-expressed in *E. coli* BL21 (DE3) in Luria Broth medium.

Cells from overnight liquid were grown to an OD_600_ of 0.4 and then induced by addition of 0.5 mmol/L IPTG. The cells were grown for an additional 3 h at 37°C. The cells were harvested by centrifugation and resuspended in a buffer consisting of 20 mmol/L Tris-HCl pH 8.0 and 50 mmol/L NaCl and sonicated. After centrifugation, the supernatant was applied to a 5-mL column of HisTrap FF resin or of Glutathione Sepharose FF resin (GE Healthcare). Following extensive washing with the binding buffer (20 mmol/L Tris-HCl pH 8.0, 50 mmol/L NaCl), samples were eluted with either buffer A (20 mmol/L Tris-HCl pH 8.0, 50 mmol/L NaCl, 300 mmol/L imidazole) for His-tagged proteins or buffer B (20 mmol/L Tris-HCl pH 8.0, 50 mmol/L NaCl, 20 mmol/L glutathione) for GST-tagged proteins. Peak fractions were pooled. Proteins were further purified by gel filtration chromatography using a Superdex-200 10/300 GL column (GE Healthcare) with 20 mmol/L Tris-HCl and 50 mmol/L NaCl, pH 8.0 as running buffer and then stored at −80°C. The His-tagged FtsZ and Ga5DH were individually used to immunize mice to produce polyclonal antibodies.

### Solid phase binding assay

Five hundred micrograms of purified FtsZ or BSA were coated in a buffer containing 14 mmol/L Na_2_CO_3_ and 36 mmol/L NaHCO_3_ overnight at 4°C onto the enzyme-linked immunosorbent assay plates. After adsorption, the wells were washed three times with phosphate-buffer saline containing 0.5% Tween-20 (PBST) to remove the excess unbound proteins. Nonspecific binding sites of the wells were blocked with 10% goat serum in PBS for 2 h at 37°C. This was followed by incubation for 2 h at 37°C with different concentrations of Ga5DH protein in PBS. The plates were then rinsed with PBST three times. Finally, the interaction of Ga5DH with FtsZ was detected with anti-Ga5DH antibodies by an enzyme-linked immunosorbent assay. The anti-Ga5DH antibodies used for the immunosorbent assay were pretreated with immobilized His-tagged enolase protein (Lu et al., 2012) to fish out anti-His antibodies. Wells coated with BSA served as negative controls. All assays were performed in triplicate.

### GST pull down assay

The pull down assay was performed with the purified GST-FtsZ or GST proteins by utilizing a modified method (Dziedzic et al., 2010). In brief, equal-molar amounts of GST-FtsZ or GST in PBS were mixed and incubated at 4°C with *E. coli* lysates containing His-tagged Ga5DH for about 4 h. Then the mixtures were allowed to bind to glutathione-Sepharose. The slurries were washed thoroughly with PBS. The bound proteins were eluted with glutathione. The samples were boiled in SDS-PAGE loading buffer for 10 min. The separated proteins were subjected to SDS-PAGE and immunoblotted. Samples were probed with an anti-His monoclonal antibody (MBL).

### Immunofluorescence microscopy

Immunofluorescence was performed as previously described with small modifications (Foulquier et al., 2011). Cells from overnight culture were diluted 100-fold into 3 mL fresh THY medium and grown at 37°C to exponential phase. The cells (1 mL) were then harvested and resuspended in 10 mL of ice-cold 80% methanol and incubated for 1 h at room temperature. The suspension was then concentrated, resuspended in 200 μL of freshly prepared 16% formaldehyde and incubated for 5 min at room temperature. Then samples were centrifuged and washed once with 1 mL of ice-cold 80% methanol. After centrifugation, cells were permeabilized at 37°C for 10 min in 20 mmol/L sodium phosphate buffer pH 6.2, 50 mmol/L sucrose, 500 mg/mL lysozyme. Cells were then washed with PBST, saturated with 200 μL of PBST-10% goat serum containing anti-Ga5DH or anti-FtsZ antibodies (dilution 1/100) and incubated overnight at 4°C. The samples were washed twice with PBST and incubated with a 1:300 dilution of the secondary goat anti-mouse antibody coupled with Alexa Fluor 488 (Santa Cruz) in the dark. DNA was visualized by treatment with the DNA fluorescent stain DAPI. For FM4-64 staining, all cells were harvested and incubated in 5 μmol/L FM4-64 in dark for 20 min. The excess dye was washed with THY medium. Finally, cells were mounted directly onto microscope slides covered with a thin film of 1.2% agar in water. Fluorescent images were acquired by laser scanning confocal microscope (Leica TCS SP2).

### Transmission electron microscopy

All samples were harvested at an OD_600_ of 0.8 and fixed with 2.5% glutaraldehyde (1 mL) followed by washing with PBS. The cells were treated with 1% osmium tetroxide for 2 h in dark. Then the subsequent dehydration steps with ethanol were carried out as follows: 50% for 15 min, 70% for 15 min, 95% for 15 min, 100% for 20 min. The samples were embedded in Spurr’s plastic and sectioned. Cell morphology was then visualized using a JEM-1400 (JEDL) transmission electron microscope.

### Scanning electron microscopy

All samples were grown in THY broth and harvested at an OD_600_ of 0.8. Cells were spotted onto polylysine coverslips followed by washing with PBS. The cells were fixed in 0.18 mol/L cacodylate buffer (pH 7.6) containing 2% glutaraldehyde. Then the subsequent dehydration steps with ethanol were carried out, passage in HMDS (1,1,1,3,3,3-hexamethyldisila zane) and finally air dried. The dried samples were covered with a 10 nm-thick gold/platinum layer. Samples were then observed with a Quanta200 (FEI) scanning electron microscope.
